# Evidence for Resident Memory T cells in Rasmussen Encephalitis

**DOI:** 10.15844/pedneurbriefs-30-3-5

**Published:** 2016-03

**Authors:** Amer Khojah, Marisa Klein-Gitelman

**Affiliations:** 1 Division of Rheumatology, Ann & Robert H. Lurie Children's Hospital of Chicago, Chicago, IL

**Keywords:** Rasmussen Encephalitis, Resident Memory T Cells, CD103 Antigen, Natalizumab, Focal Cortical Dysplasia

## Abstract

Investigators from University of California Los Angeles studied the presence of different T cell subset population in the brain tissue of 7 patients with Rasmussen encephalitis, a rare neuroinflammatory disorder characterized by intractable seizures and usually associated with progressive hemi cerebral atrophy, who underwent brain surgery and compared them to patients with focal cortical dysplasia.

Investigators from University of California Los Angeles studied the presence of different T cell subset population in the brain tissue of 7 patients with Rasmussen encephalitis, a rare neuroinflammatory disorder characterized by intractable seizures and usually associated with progressive hemi cerebral atrophy, who underwent brain surgery and compared them to patients with focal cortical dysplasia. Clusters of T cell (mostly CD8) were found at the gray and white matter junction in patients with Rasmussen encephalitis. The majority of these CD8 T cell express CD103 and CD 69, markers of tissue resident memory T cells, irrespective of the time between surgery and the onset of seizure. This finding suggests that the immune response occurs very early in the course of the disease as early as 3 months. In contrast, T cells in patients with focal cortical dysplasia were found near the blood vessels and less than 10% of these cells expressed CD103. However, the percentage of CD103 + CD 8 T cell correlated with the duration of illness suggesting that inflammation occurs later in the disease course and it is accumulative over time. [1]

COMMENTARY. Naive T cells undergo rapid proliferation phase (clonal expansion) after interacting with antigen on antigen presenting cell. The majority of these activated T cells differentiate into different effectors cells based on the cytokine milieu and travel to the affected organ by expressing different tissue homing molecules. After successful elimination of the pathogens, these cells die by apoptosis (contraction phase). Small percentage of naive T cells differentiate to memory T cells, which live for long period of time and protect the body from future infections. There are 3 distinct types of memory T cell: 1) Central memory T cells (TCM) which express CCR7 and home to secondary Lymphoid organs. 2) Effectors memory T cells (TEM) which are shorter-lived but more active cells. These cells are able to go to blood stream and different tissues. 3) Resident memory T cells (TRM), a newly recognized type of memory T cells that express CD103 and/or CD69. These cells reside in the affected tissue even after the pathogen is cleared and do not circulate in the blood stream [2]. Beside the role of these cells in clearing pathogens, there is growing body of evidence of their role in organ-specific autoimmunity like psoriasis and Crohn's disease [2]. TRM cell has been found in the junction between gray and white matter in a mouse model of relapsing - remitting CNS disease (multiple sclerosis) suggesting that these cells can present in the tissue even in the absence of previous infection [3]. Natalizumab, a monoclonal antibody that blocks the T cell's ability to cross the blood brain barrier by targeting alpha-4 integrin and an FDA approved treatment of multiple sclerosis, has been used with good success in a patient with Rasmussen encephalitis [4]. Unfortunately, the use of Natalizumab is associated with increased risk of PML (progressive multifocal leukoencephalopathy) from reactivation of JC virus infection. For patients with inflammatory bowel disease, this risk can be minimized by using Vedolizumab, alpha-4 beta-7 integrin antibody which is gut homing molecule and does not interfere with CNS lymphocytes trafficking [5]. The investigators in this study showed the presence of memory resident T (TRM) cell in the brain tissue of Rasmussen encephalitis patients at early stages of the disease. This finding expands our knowledge about Rasmussen encephalitis, a poorly understood inflammatory disease and gives insight about new therapeutic approaches, such as using Natalizumab to slow the disease progression and hopefully eliminate the need for surgery in patients with Rasmussen encephalitis.
